# Gut Microbiome and the Response to Immunotherapy in Cancer

**DOI:** 10.15190/d.2018.4

**Published:** 2018-09-30

**Authors:** Andreea Lucia Stancu

**Affiliations:** Department of Dermatology, Brigham and Women’s Hospital, Harvard Medical School, Harvard Institute of Medicine, 4 Blackfan Circle, Boston, MA, 02215, USA. E-mail: astancu@bwh.harvard.edu

**Keywords:** Cancer, immunotherapy, microbiome, response to therapy, PD-1, PD-L1.

## Abstract

Recent studies indicate that the composition of gut bacteria can influence the effectiveness of certain cancer immunotherapy drugs and that modulating the gut microbiome may expand the pool of patients benefiting from cancer immunotherapies. Checkpoint blockade therapy has been effective on several types of malignancies (e.g. melanoma, lung cancer, kidney cancer). However, the number of patients that do not respond, or only partially respond, to cancer immunotherapy is high. Recently, several human and mouse studies have shown that gut microbiome may be a significant determinant of the response to cancer immunotherapy. This review focuses on the recent advances in our understanding of the interaction between human gut microbiome and response to immunotherapy in cancer. The gut microbiome may serve as a theranostic biomarker, by acting both as a useful prognostic biomarker and a target in cancer therapy.

## 1. Introduction

Immune checkpoint therapies have enabled a breakthrough in the therapy of the hematologic and solid metastatic malignancies^1-4^. Administration of monoclonal antibodies unleashes a T lymphocyte-mediated immune response by inhibiting the interaction of T-cell suppressing receptors with their corresponding ligand on tumor cells^2^. In particular, immune checkpoint inhibitors that target programmed cell death protein 1 (PD-1) and its ligand PD-L1, have to date been approved by the U.S. Food and Drug Administration (FDA) for the use in the treatment of patients with 10 distinct cancer types^5^.

In anti-tumor therapy, specific CD8+ cytotoxic T lymphocytes (CTL) are generated, recognize specific tumor antigens and induce caspase-dependent cell death in tumor cells^6,7^. However, tumor cells can utilize a wide range of immune escape strategies. An important escaping mechanism is the one exploiting the PD-1 - PD-L1 interaction, in which CTLs are inhibited through PD-1 receptor on their surface by the PD-L1 ligand, found on tumor or other cells. For example, it was shown that PD-L1 expressing cancer cells induce caspase-dependent cell death of co-cultured activated CTLs and an anti-human PD-L1 monoclonal antibody can block this interaction and its effects. Other studies replicated these results in mouse models^6,8-10^. Although most normal tissues do not express detectable PD-L1 levels on their cellular surface, PD-L1 is highly expressed in tumor cells and some immune/non-immune cells in tumor environment^11-19^. Moreover, certain cytokines, such as TNF-alpha, IL-4, IL-10 and IFN-gamma play an important role in increasing the expression of PD-L1^6,20-22^. PD-1 interaction with its PD-L1 ligand leads to suppression of the effector T cells, by inducing T cell apoptosis, anergy and/or exhaustion^6,20,23^*. *Moreover, the phagocytic activity of macrophages against tumor cells can also be suppressed by PD-1 - PD-L1 interaction^6,24^*. *All these results and others not mentioned here led to a significant number of studies focusing on disrupting this interaction for cancer treatment^6^.**

Many clinical trials are now underway, testing monoclonal antibodies against PD-L1 or PD-1, as a cancer immunotherapy strategy for a variety of malignancies. These treatments may work both by disrupting the immunological-related pathways, but also by disrupting intracellular tumor signaling pathways^25^. Currently, there are at least 5 monoclonal antibodies targeting PD-1 or PD-L1 approved by the FDA as cancer immunotherapy^6^.

Responses to immunotherapy targeting PD-1 or PD-L1 are often heterogeneous and not durable. Primary resistance, observed in the majority of the cases, has been associated with low mutational burden, poor intrinsic antigenicity of tumor cells, defective antigen presentation during the priming phase, functional exhaustion of tumor-infiltrating lymphocytes and local immunosuppression by extracellular metabolites^6,8-11,26,27^. Thus, different approaches to optimize the efficacy of cancer immunotherapy are in high demand.

**Figure 1:**
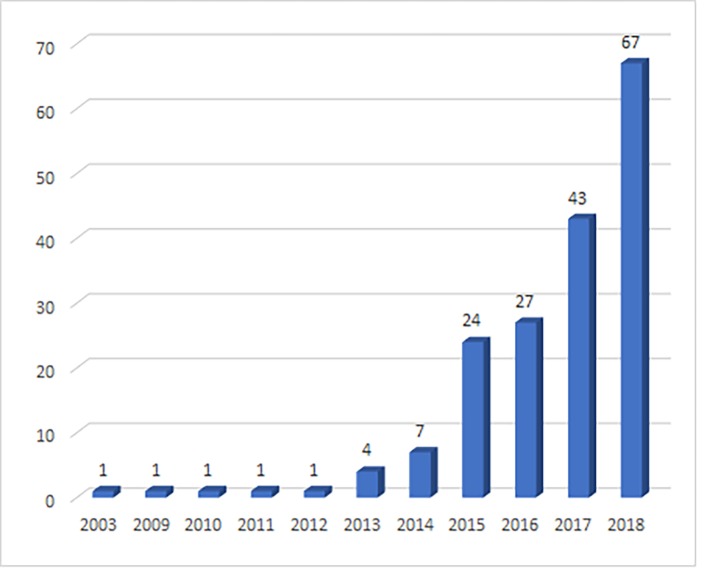
Figure 1. Exponential growth of the number of articles published between 2003-2018 related to the “cancer immunotherapy” and “microbioma”. This graph is based on the PubMed publications data. Data for 2018 is collected for January-September 2018.

Recent studies suggest that modulation of the gut microbiota is a promising approach. Several human and mouse studies have shown that gut microbiome may be a significant determinant of the response to cancer immunotherapy^6,15,17-19,28-35^. The number of studies investigating microbiome’s relation with the cancer immunotherapy is growing exponentially (**Figure 1**). Thus, we and others think that the gut microbiome may serve as a theranostic biomarker, by acting both as a useful prognostic biomarker and a target in cancer therapy.

## 2. Role of microbiota in immune checkpoint therapy

Stem cell transplant can be considered one of the first immunotherapy strategies for cancer treatment (e.g. transplant for leukemia treatment). In recent years, an exponential number of studies^30,35^ initiated new immunotherapeutic approaches, which were initially demonstrated in mouse models with many of them (e.g. anti-PD-1 or anti-PD-L1 treatments) being validated in humans^30,35^.

Although not considered immunotherapy, the response to the standard chemotherapy treatment is at least in part based on the activation of the immune system. Over 10 years ago, Paulos et al. observed that antibiotics inhibited anti-cancer activity in melanoma, a murine model of adoptive cell therapy, while translocation of bacteria from the gut helped by initiating a TLR4-dependent immune response^36^. Subsequent studies confirmed these findings by using platinum and cyclophosphamide-based therapy^34,37^.

The role of gut microbiome as a response to the immune checkpoint blockade was similarly initiated in mouse models, showing that gut bacteria influences the response to cancer immunotherapy (targeting CTLA-4 and PD-1)^30,35^*.* Many of the patients do not respond to the treatment with immune checkpoint inhibitors. Interestingly, oral administration of certain types of Bacteroides (Bacteroides fragilis together with Burkholderia cepacia or Bacteroides thetaiotaomicron) stimulated anti-CTLA-4 immunotherapy^35^. Many other studies were subsequently published showing that gut bacteria is associated and/or modulates the resistance to immunotherapy, such as anti-PD-1 or anti-PD-L1 treatments^6,15,17-19,28-34^.

Of note, microbiota can be transplanted to other mice and the beneficial effects are transmitted to the new host. Supplementation with a probiotic orally administered containing Bifidobacterium re-sensitized the tumors to anti-PD-L1 therapy in mice, by promoting DC maturation and an increase in anti-tumor CD8+ CTL activity^33^.

In the past two years, a significant number of bacteria were determined to modulate anti-PD-1 or anti-PD-L1 therapy in a wide range of malignancies, such as melanoma, lung cancer, kidney cancer. A summary of the most important bacterial candidates is presented in **Table 1**. Notably, few of them (such as Bifidobacterium) were confirmed in multiple studies to be present in higher abundance in responders to immunotherapy and that mice that received the bacteria species Bifidobacterium have a marked increase in anti-tumor T cell responses^30^.

**Table 1:** Examples of bacteria as potential modulators of the anti-PD-1 immunotherapy in cancer (their modulatory mechanisms and effects have to be further investigated). Ref. – Reference(s).

Bacteria (Gut)	Effect	Ref.
Ruminococcaceae family	Higher abundance in responders to anti-PD-1 therapy (melanoma); also, higher diversity of bacteria observed	^33^
Faecalibacterium genus	Higher abundance in responders to anti-PD-1 therapy (melanoma); also, higher diversity of bacteria observed	^33^
Bifidobacterium longum and Bifidobacterium adolescentis	Higher abundance in responders to anti-PD-1 therapy (metastatic melanoma)	^29,30^
Collinsella aerofaciens	Higher abundance in responders to anti-PD-1 therapy (metastatic melanoma)	^29^
Enterococcus faecium	Higher abundance in responders to anti-PD-1 therapy (metastatic melanoma)	^29^
Klebsiela pneumoniae	Higher abundance in responders to anti-PD-1 therapy (metastatic melanoma)	^29^
Veillonella parvula	Higher abundance in responders to anti-PD-1 therapy (metastatic melanoma)	^29^
Parabacteroides merdae	Higher abundance in responders to anti-PD-1 therapy (metastatic melanoma)	^29^
Lactobacillus sp.	Higher abundance in responders to anti-PD-1 therapy (metastatic melanoma)	^29^
Akkermansia muciniphila	Higher abundance in responders to anti-PD-1 therapy (lung and kidney cancers); exposure to antibiotics decreases response	^31^
Ruminococcus obeum	Higher abundance in non-responders to anti-PD-1 therapy (metastatic melanoma)	^29^
Roseburia intestinalis	Higher abundance in non-responders to anti-PD-1 therapy (metastatic melanoma)	^29^

The mechanisms through which some bacteria enhance anti-tumor immunotherapy are not yet clearly defined. Recent studies suggest that tumor antigens are similar to those found on infectious pathogens^38,39^***.***Other studies suggest that the metabolic effects are involved or that the microbiota determines “the tonus of the immune reseponse”^39,40^.Thus, further studies are required to elucidate these mechanistic aspects.

## 3**. Microbial intervention as adjuvant therapy for immunotherapy in cancer**

Considering the exponential increase of the scientific community’s interest and the exciting already published studies, the involvement and potential modulation of gut microbiota may represent an important adjuvant in cancer immunotherapy in the near future.

Although the exact molecular mechanisms of the microbiota interactions with cancer therapies are not completely understood, several clinical trials are already ongoing (**Table 2**). For example, at least 3 observational studies (NCT02960282, NCT03643289, NCT0368834) and one interventional (NCT03686202) are now recruiting patients (**Table 2**). In the first mentioned trial, the gut microbiome will be investigated in the fecal samples from patients with metastatic cancer undergoing chemotherapy and immunotherapy. In a second clinical trial, the gut microbiome diversity will be assessed with metagenome sequencing before and after immunotherapy, in order to predict the response to immunotherapy for melanoma. The third trial will investigate the nasal, oral and fecal microbiome, and correlate the data with treatment response and toxicities of immunotherapy, in lung cancer and other malignancies. The NCT03686202 interventional clinical trial (phase 1) will assess the safety, tolerability and engraftment of Microbial Ecosystem Therapeutics (MET-4) strains when given in combination with immunotherapy, in solid tumors. Other clinical trials aim investigating the dietary, other lifestyle or environmental factors in relation to cancer therapy, or the direct use of live genetically modified bacteria as an immunotherapy treatment (**Table 2**).

**Table 2:** Clinical trials evaluating microbial role/intervention as immunotherapy modulator in cancer ***(based on ClinicalTrials.gov, website maintained by the National Library of Medicine, at the US National Institutes of Health (NIH)*** **

ClinicalTrials.gov Identifier	Name of the study	Conditions	Comments	Status
NCT02960282	Gut Microbiome in Fecal Samples from Patients with Metastatic Cancer Undergoing Chemotherapy or Immunotherapy	Colorectal Cancer (stage 4)	Observational study; Biospecimen Collection; Laboratory Biomarker Analysis	Recruiting
NCT03643289	Predicting Response to Immunotherapy for Melanoma with Gut Microbiome & Metabolomics	Melanoma (stage 4)	Observational study; Assess the gut microbiome diversity with metagenome sequencing before and after immunotherapy	Recruiting
NCT03688347	Microbiome in Lung Cancer and other Malignancies	Lung cancer, other cancers	Observational study; Assess the nasal, oral and fecal microbiome, and correlate the data with treatment response and toxicities of immunotherapy	Recruiting
NCT03686202	Feasibility Study of Microbial Ecosystem Therapeutics (MET-4) to Evaluate Effects of Fecal Microbiome in Patients on Immunotherapy	Solid tumors	Interventional (Phase 1); Assess the safety, tolerability and engraftment of Microbial Ecosystem Therapeutics (MET-4) strains when given in combination with immunotherapy	Recruiting
NCT02592967	Safety & Immunogenicity of JNJ-64041757, Live-attenuated Double-deleted Listeria Immunotherapy, in Subjects with Non Small Cell Lung Cancer	Non-small cell lung cancer (NSCLC) (stages 3B and 4)	Interventional (Phase 1); Assess the JNJ-64041757, a live attenuated double deleted (LADD) Listeria monocytogenes as single immunotherapy	Active
NCT02625857	Safety & Immunogenicity of JNJ-64041809, a Live Attenuated Double-deleted Listeria Immunotherapy, in Participants with Metastatic Castration-resistant Prostate Cancer	Prostate cancer, castration-resistant	Interventional (Phase 1); Assess the JNJ-64041809, a live attenuated double deleted (LADD) Listeria monocytogenes as single immunotherapy	Completed
NCT02843425	The Beans to Enrich the Gut Microbiome vs. Obesity's Negative Effects (BE GONE) Trial	Colorectal cancer Prevention	Interventional; Assess the fiber supplementation -induced shift in bacterial populations, through the addition of a half cup of beans/day into the normal diets of cancer patients	Recruiting

Diet may serve as an important tool for modulating the microbiome in relation to the anti-cancer immunotherapy. The main function of microbiota is to help in food digestion and producing valuable factors (e.g. vitamin K), that the host can’t produce itself^41^. Dietary variation modifies the microbiome composition. Several proof-of-concept studies demonstrated how dietary modulation can be employed as a strategy to influence the gut microbiome and immune health^42,43^. Dietary fibers have been previously established to have an important influence on the gut microbiome composition^44^*. *The NCT02843425 (“BE GONE”) interventional clinical trial assesses the fiber supplementation-induced shift in bacterial populations, through the addition of a half cup of beans/day into the normal diets of cancer patients (colorectal cancer). Additional clinical trials investigating the role of dietary supplements and other factors on the microbiota/microbiome in the context of cancer are underway (NCT02079662, NCT03072641, NCT02928523, NCT01895530, NCT03353402, NCT03358511)^33^**. **Although, all these studies are just starting, they will provide critical information on how diet, lifestyle and environmental factors modulate the gut microbiome, provide new prognostic biomarkers and modulate the patient outcome.

## **4.** **Future directions**

In the very recent few years, we have gained significant insights into the role of gut microbiome in cancer therapy, with a special focus on immunotherapy. The number of published studies is growing exponentially, and the first clinical trials are now ongoing. However, the exact mechanisms, involved bacteria and utility in various types of malignancies remain to be further investigated and understood.

In order to efficiently tackle these objectives, one has to use the optimal methods available. For example, selection of the sequencing methods for the microbiome and reference databases is very important^33^. Since reproducibility is often brought into question, it is important to properly set-up and execute these studies^45^.

Further investigations should determine the bacterial combination pools needed to facilitate the response to cancer immunotherapy in different types of malignancies and how we can develop or induce the development of such combinations. The outcome should be carefully investigated in clinical trials. Moreover, the role of other influencers, such as medications (drug treatments for other conditions, antibiotics, probiotics etc), diet, metabolic changes (e.g. exercise) and other internal conditions (e.g. mental health) and external factors have to be thoroughly investigated.

Taking advantage of the prognostic and modulatory role of the microbiome in cancer immunotherapy in particular, and cancer therapy in general, to enhance the anti-tumor immunity and immune surveillance, may be one of the dominant strategies in cancer therapy in the future.
